# Virus-like Particles as Nanocarriers for Intracellular Delivery of Biomolecules and Compounds

**DOI:** 10.3390/v14091905

**Published:** 2022-08-28

**Authors:** Junyao He, Linying Yu, Xiaodi Lin, Xiaoyan Liu, Yanming Zhang, Fan Yang, Wen Deng

**Affiliations:** College of Veterinary Medicine, Northwest A&F University, Xianyang 712100, China

**Keywords:** virus-like particles, nanocarrier, intracellular delivery, cargo loading strategies, delivery vehicles

## Abstract

Virus-like particles (VLPs) are nanostructures assemble from viral proteins. Besides widely used for vaccine development, VLPs have also been explored as nanocarriers for cargo delivery as they combine the key advantages of viral and non-viral vectors. While it protects cargo molecules from degradation, the VLP has good cell penetrating property to mediate cargo passing the cell membrane and released into cells, making the VLP an ideal tool for intracellular delivery of biomolecules and drugs. Great progresses have been achieved and multiple challenges are still on the way for broad applications of VLP as delivery vectors. Here we summarize current advances and applications in VLP as a delivery vector. Progresses on delivery of different types of biomolecules as well as drugs by VLPs are introduced, and the strategies for cargo packaging are highlighted which is one of the key steps for VLP mediated intracellular delivery. Production and applications of VLPs are also briefly reviewed, with a discussion on future challenges in this rapidly developing field.

## 1. Introduction

The delivery of genes and their products (nucleic acids and proteins) into cells or tissues covers a broad spectrum of applications with respect to vaccines against viruses or cancers, gene therapy and diagnostic imaging [1,2,3,4]. Diverse delivery methods are established by using biological, chemical and physical approaches to introduce these biomolecules into target cells [4]. Cargo delivery by nanocarriers, such as inorganic nanoparticles and lipid-nanoparticles, have been widely used in recent years. Among these nanocarriers, virus-like particles (VLP) are biologically derived nanostructures that are of special interests as they have unique features and advantages in comparison to other methods. 

VLPs are natural or artificial nanostructures mimicking viruses but without enough viral genetic materials to support replication. VLPs can be naturally produced as defective virus and are observed in normal virus infection [5]. The fact that some viral structural proteins can be recombinantly expressed and self-assemble to virus-like structures enables artificial design and production of VLPs. Therefore, VLPs with different features, either enveloped or without envelope, have been engineered for a variety of purposes. With structures similar to normal virus, the VLPs generally have better immunogenicity than soluble protein monomer antigens. This makes VLPs a promising strategy for development as preventive vaccines for infectious diseases and therapeutic vaccines against different types of tumors [6].

The three-dimensional structure and cell invasion property of VLPs also makes them an excellent candidate for intracellular delivery of biomolecules. Proteins, nucleic acids or chemical compounds may be carried by the VLP and delivered into different types of cells. The cargo molecules are protected by the nanocarrier from degradation or undesired binding in vivo, and genetic engineering of the VLPs can guide the carrier to specific cells or organs, achieving targeted delivery of cargo. Moreover, diverse cargo loading methods have been developed, some of which are able to release the cargo in a controlled way, enabling precise delivery of biomolecules. With the increasing advances in gene therapy, genome editing and precise drug administration, development and application of VLPs for intracellular delivery of diverse biomolecules and drugs have grown extensively [7]. This review focusing on VLPs as nanocarriers is presented to help the community understand recent progress and potential challenges in VLPs for intracellular delivery.

In this review, we first describe the general progress on delivery of biomolecules and drugs by VLPs, then highlight the cargo loading strategies since loading is one of the key steps for molecule delivery. Next, frequently used VLPs derived from multiple virus species will be discussed more specifically on their properties, production and applications, and finally we provide a short future perspective for VLPs as delivery tools.

## 2. VLP Involvement in Intracellular Delivery

### 2.1. Strategies for Intracellular Delivery

At present, common methods for biomolecule or compound delivery include physical methods, chemical methods and virus-based delivery systems. Physical methods deliver biomolecules into cells either by physical force (e.g., electroporation, acoustic perforation and magnetic transfection) [8,9,10,11,12] or by using mechanical tools to send materials directly into the cell (e.g., microinjection and gene guns) [13,14], preferably for applications in vitro. Chemical methods use chemicals or biochemicals, such as liposome, polymers, cell-penetrating peptides, exosomes or inorganic nanoparticles (silica or carbon skeleton), and virus-like particles [15,16,17,18,19,20], to mediate cargo including proteins and nucleic acids to pass the cell membrane. These methods have been widely used in vitro and in vivo. Chemical methods, which attract much attention in the fields of gene therapy and drug delivery, have some unique advantages such as low immunogenicity, high safety, the feasibility of artificial synthesis and large-scale production. However, some challenges remain, for example, nanomaterials must be chosen carefully to avoid toxicity in vivo, targeted delivery of the cargo is rather challenging, and chemical nanocarriers are difficult to escape and release from endosomes [19].

**Figure 1:**
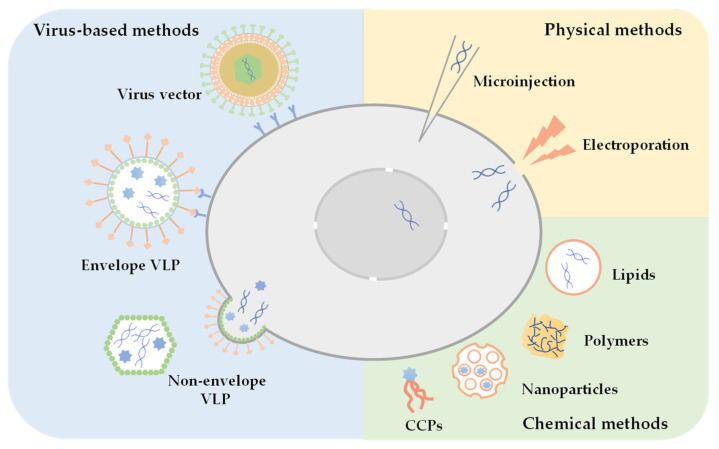
Methods for intracellular delivery. Physical methods such as electroporation and microinjection, chemical methods such as liposome, nanoparticles, etc., and virus-based methods like infectious viral vectors and VLPs, are the major categories of intracellular delivery methods.

Viruses are natural vectors that deliver the viral protein and genome into permissive cells; therefore, viral vectors were initially developed for delivery of foreign genes. Lentivirus, adenovirus and adeno-associated virus vectors are most frequently used for gene therapy [21], but inevitably, viral vectors for gene delivery are generally considered potentially risky and carry limited exogenous substance [22]. Therefore, a special type of virus vectors, VLPs, are developed for delivery of both nucleic acids and proteins. VLPs have similar advantages as virus vectors but with better biosafety since VLPs lack viral genetic materials. VLPs have nanostructures mimicking virions, therefore they can be internalized by cells and disassemble in the cytosol to release the cargo. VLPs can also be genetically modified for targeted delivery. These advantages make VLP a popular nanocarrier for intracellular delivery of different biomolecules. Figure 1 displays different vectors for delivery of cargo.

### 2.2. VLP-Mediated Intracellular Delivery

#### 2.2.1. VLPs for Protein Delivery 

Proteins participate in almost all biological activities and are begin explored as important therapeutics in recent years. It is difficult for proteins to enter the target cells directly because large molecular proteins are challenging to cross the cell membrane barrier and might elicit immune responses in vivo. Several delivery methods, such as cell-penetrating peptides (CPPs) [23], extracellular vesicles (EVs) [24], polymeric micelles [25,26], and liposomes [27,28], have been used to deliver foreign proteins into cells, but typically lack the capability of targeting specific cells [17]. Therefore, VLPs which feature natural properties like biocompatibility, bioavailability, modifiability of coat proteins for tropism, and the capacity of active entry into cells have great potential for protein delivery. 

VLPs developed from both enveloped viruses such as murine leukemia virus (MLV), avian sarcoma leukosis virus (ASLVs), paramyxovirus (PIV5, Nipah) and human immunodeficiency virus-1 (HIV-1) and non-enveloped viruses like bluetongue virus (BTV) have been used for the targeted delivery of proteins [29,30,31,32,33,34,35,36,37]. Cargo proteins are fused to or inserted into VLP component proteins with a protease-cleavable linker, then are transported and released into the target cells or cell nuclei with the help of viral protease or by other mechanisms [38,39]. For proof-of-concept, fluorescent proteins or enzymes (GFP or Rluc) were carried by VLPs, and later toxic proteins, transcription factors, recombinase (Flp, Cre), and HSV-1 thymidine kinase were transported into targeted cells, with their original activities fully maintained, demonstrating a superior strategy for intracellular delivery of functional proteins [29,30,31]. Limited by space and size of the VLP, relatively small proteins were packaged and delivered at first. In recent years, with the rapid development of gene editing technology for therapeutic purposes, delivery of proteins or fusion-proteins with large molecular weight (such as Cas9 proteins and dCas9-base editors) have been increasingly interesting to researchers. In 2019, Mangeot et al. successfully packaged the spCas9 or dCas9-VPR fusion protein (up to 244 kDa) together with sgRNA into MLV VLPs, later also with a donor ssDNA for knock-in of foreign genes. The VLPs carrying these huge proteins have slightly larger sizes than normal MLV VLPs, and successfully mediated genome editing or gene activating in primary cells and also in mouse models, demonstrated the potential for package and delivery of huge proteins and protein-nucleic acid complexes with MLV VLPs [32]. Recently, Banskota et al. systematically optimized the cleavable linker between the cargo and MLV gag protein to enhance cargo release, tested the NES/NLS localization signal peptide and the stoichiometry of packaging plasmids for correct localization and cargo packaging efficacy, respectively. Eventually, they developed base editor engineering VLPs (BE-eVLPs) which could deliver base editors fused to Cas9 (about 184 kDa) into cell nuclei to modify genes in cultured primary cells or CNS cells in animal [33]. Protein delivery via VLPs is summarized in Table 1.

There are, however, still several bottlenecks for VLPs to deliver proteins, such as the limitation of the amount of loading cargo, correct release of proteins inside the target cells, precise localization of cargo at the cytoplasm and cell nucleus, as well as engineered targeting specificity in vivo. Further improvements would greatly help the applicability of VLP for protein delivery.

#### 2.2.2. VLPs for Nucleic Acid Delivery

Nucleic acids are desired cargo for therapeutic applications. However, direct delivery of nucleic acids is limited by the physical and chemical features that they are negatively charged, typically with large linear structures, and poor stability for RNA molecules. Virions are naturally nanocarriers for viral genomic DNA or RNA, therefore, VLPs could be engineered conveniently for intracellular delivery of nucleic acids. 

Delivery of DNA molecules goes back two decades ago when non-replicative adenovirus, which indeed is a VLP, was tested together with replicative adenovirus vectors to deliver DNA encoding human p53 protein into tumor cells for therapeutic purposes [59]. Since then, VLPs derived from different virus species have been developed for delivery of DNA encoding a variety of functional proteins, some of which have been approved for clinical use. For example, non-replicative AAV-mediated delivery of nucleic acids encoding LPL, RPE65 and SMN1 have been approved for treatment of genetic diseases in the EU or US [60]. 

Although therapeutic DNA delivery was developed first, VLPs are favored for RNA delivery in recent years as RNA in general has better biosafety than DNA, and different types of RNAs have been delivered with VLPs. 

mRNAs can be delivered into the cytoplasm directly and translated into functional proteins without the risk of genome integration. In addition, technologies for in vitro transcription (IVT) or chemical synthesis of mRNA are mature and cost-effective and suitable for large-scale mRNA production. However, the mRNA is easily degraded by RNases due to its intrinsic biochemical nature, thus efficient delivery of mRNA is rather challenging and improved delivery methods are required for broad applications of mRNA therapeutics. The VLP can protect mRNA from the environment and stabilize mRNA for better delivery efficiency, and VLPs derived from bacteriophage Qβ were shown to encapsulate mRNA fusing to the Qβ stem-loop RNA structures [61]. In addition to the VLP being derived from virus, the artificial coat proteins which include an oligolysine (K12), silk-like midblock S10 as well as a hydrophilic random coil polypeptide, enables self-assembly into rod-shaped VLPs with incorporation of about one to five mRNA molecules [43]. While the C-terminal oligolysine (K12) domain can bind to the nucleic acid, the silk-like midblock S10, a 10-fold tandem repeat of the octapeptide, can stack into a rigid filament and form the scaffold of the rod-shaped VLPs. A long hydrophilic random coil block C plays a functional role in maintaining the stability of VLPs and shielding mRNA molecules [44]. However, this VLP has very low transfection rate and devoid of a cell-targeting functionality [43], therefore, further improvement on cellular uptake and endosomal release are required. 

In addition to the regular VLP derived from active virus, the Liu lab developed a new RNA delivery strategy which uses mouse endogenous retroviral gene products to assemble virus-like particles. The system can transfer mRNA of interest such as Cre mRNA and Cas9 mRNA into targeted cells [45]. The researchers screened a series of conserved retroelement genes suitable for mRNA delivery and eventually found that the richest protein within the VLP components was PEG10 derived from mouse endogenous virus, which is homologous with retroviral gag protein. Just like gag protein, PEG10 can package the mRNA encoding itself and assemble to virus-like structures then be secreted by extracellular vesicles [62,63]. Thereby, VLPs were produced by co-transfecting a plasmid encoding PEG10 protein and a plasmid for transcribing mRNA of interest flanked by PEG10 5’UTR and 3’ UTR which is necessary for packaging, with the vesicular stomatitis virus envelope protein (VSV-G) expressed to facilitate cell entry of the VLP. This complete endogenous delivery system may minimize immunogenicity of the delivery vector and could be an ideal method for gene therapy.

Artificially synthesized small interfering RNAs (siRNAs) or microRNA (miRNA) can destabilize targeting mRNA and exert gene silencing effects [16], thus are widely applied to gene therapy, autoimmune diseases, and cancer treatments. Just as mRNA, siRNA also poses a serious challenge for delivery, such as easy degradation by RNases, inefficient systemic delivery and failure to cross the cellular barriers [64,65]. VLPs, as the nanocarriers for siRNA delivery, may overcome these issues, and VLPs have been established for siRNA delivery both in vivo and in vitro [66,67,68]. Suffian et al. reported that engineered HBc-VLPs targeting HER2 expressing on the surface of cancer cells can carry siRNA to knock down the polo-like kinase 1 (PLK1) gene in cultured cancer cells, thereby inducing death of the tumor cells [47]. However, the knockdown effect of PLK1 gene using HBc-VLPs-siPLK1 is weak in mice, showing a declined protein amount of only about 10%, which may be because of limited amounts of siRNA loaded into VLPs and low delivery efficacy when injecting in vivo. In addition, AAV2-VLP is an example of an siRNA transferring vector for breast cancer treatment. A polymer modified VLP, i.e., PEI-AAV2-VLPs, protected siRNAs from the degradation of RNases and effectively transfected siRNAs in cancer cells [46]. Bacteriophage PP7 and MS2 originated VLPs can effectively carry microRNA into the tumor cells or tissues and subsequently suppressed its targeting gene [55,69], demonstrating successful delivery of small RNA into mammalian cells by phage VLPs.

In some cases, both mRNAs and noncoding RNAs need to be co-delivered via VLPs. To achieve Cas9-sgRNA mediated genome editing, Yadav et al. adopted the strategy of VLPs co-packaging Cas9 mRNA and sgRNA [48]. Cas9 mRNAs or sgRNAs were fused to an RNA aptamer (e.g., com RNA) which could be recognized by aptamer binding protein (ABP, e.g., control of mom, Com protein) [70,71,72,73]. The ABP was fused to LV’s nucleocapsid (NC), and both the Cas9 mRNA and sgRNA could be recognized via ABP-aptamer interaction and packaged into VLPs by the NC protein. The proportions of unmodified NC and ABP-modified NC proteins were optimized to balance VLP production and foreign RNA packaging. It is clear that Cas9 mRNA and sgRNA all-in-one VLP delivery into cells can consistently express more Cas9 proteins in the cytoplasm, and theoretically mRNA delivery via VLP resulted in more Cas9 proteins than direct delivery of Cas9 protein [74]. 

Ribonucleoproteins (RNPs) are complexes formed by protein and RNA, which exist widely in cells and play many different roles. The nuclease active Cas9 in CRISPR-Cas systems is a RNP composed of the Cas9 protein and sgRNA, and how to effectively deliver the RNP for genome editing or targeting is one key issue for gene therapy of genetic diseases. Currently, retroviral vectors and bacteriophage-like particles have been modified for delivering Cas RNPs. The Cas RNPs could be packaged with direct fusion method and physical interaction method [49]. More specifically, Cas protein could be fused to the C-terminus or N-terminus of the viral Gag proteins [32,49,75], and sgRNA could bind and package together with the Cas protein simultaneously. For example, to inhibit infection of dengue virus (DNV), Singsuksawat et al. produced VLP pseudotyping by VSV-G for targeting the primary human cells to deliver Cas13b RNPs which target and cut the RNA genome of DNV [49]. They successfully delivered the RNP into primary cells and virus inhibition effects were observed, proving the feasibility of the RNP delivery approach. In addition, P22-VLP from phage can encapsulate Cas9 protein and sgRNA by genetic modification, showing the potentiality for delivery of RNPs with phages [57].

#### 2.2.3. VLPs for Compound Delivery

While most chemical drugs, like small compounds, are permeable to cells and thus can diffuse into cells randomly, it is desired to concentrate the drug in its target cells more specifically so the drug efficacy can be enhanced and potential side effects can be reduced. For some drugs, such as cell-killing drugs for tumor therapy, precise administration of the drug is required to kill the tumor cells specifically. VLPs are one of the ideal choices for drug delivery and has many advantages. First, VLPs could be engineered for targeted intracellular delivery of the drug, accumulating active drugs within the desired tissues, thereby promoting treatment effects [76]. Second, VLPs encapsulate the drug molecules to protect them from degradation or dilution within the blood, and could achieve controlled-release of the drug in target tissues or cells [77]. Third, in comparison to other types of nanocarriers, the endosome escape efficiency of VLPs is much higher than that of liposomes and inorganic nanoparticles, and VLPs have better biocompatibility and biodegradability than most chemical nanocarriers [78].

With these advantages, drugs for tumor therapy and antimicrobial infection have been delivered into cells with VLPs via different targeting strategies. In spite of the natural tropism of VLP, the function of cell targeting is typically achieved either by chemical modification on its surface to display ligand or antibody, or by genetic modification to insert peptides into the coat proteins [6]. VLP from the foot-and-mouth disease virus (FMDV), which was loaded with the anticancer drug doxorubicin (DOX), can deliver the drug into HeLa cells via the RGD motif binding to the integrin receptor sitting on the surface of tumor cells, and the anti-tumor experiment in mice demonstrated the high tumor killing efficacy of DOX-loaded VLPs [50]. Likewise, the rotavirus capsid protein VP6 self-assembled VLP, which carried DOX by covalent conjunction also showed promising delivery of the drug into cultured cells. Modification of the VLP with lactobionic acid (LA) which binds to asialoglycoprotein receptors (ASGPRs) on the surface of the hepatoma cell line, showed specific delivery of the drugs to the HepG2 cells [51,79]. In addition, targeted delivery of the drug could be guided by single chain fragment variable (scFv) modified VLPs. scFv derived from humanized CC49 antibody (hCC49) was displayed on the Rous sarcoma virus (RSV) derived VLP, and this engineered drug delivery VLP could deliver DOX to kill human colon carcinoma cells in vitro specifically without observed adverse effects to co-cultured 293T cells [52]. Cell targeting delivery of drugs could also be achieved by insertion of polypeptides into the surface loop of capsid proteins. For instance, porcine parvovirus capsid protein VP2 (PPV VP2) can self-assemble to VLP, and a 12-residue peptide (TWYKIAFQRNRK), termed as the TK peptide, was inserted into the loop region of VP2, and this VLP could specifically target and deliver drugs towards Caco-2 cells and HUVEC cells by binding to the integrin α6β1 receptor and integrin αvβ3 receptor, respectively [53]. Similarly, MS2-and Qβ-VLP were modified with a targeting peptide, SP49 (SFSIIHTPILPL), on its surface for delivery of chemotherapeutic drugs. SP49-modified VLPs exhibited high affinity and specificity towards hepatocellular carcinoma (HCC) cells but a low cytotoxicity to normal hepatocyte [54]. Of course, the adenoviral dodecahedron (Dd) vector displays a natural tropism in tumor hepatic cells as it retains the affinity for αvβ3 and αvβ5 integrins and heparan sulfate [80,81,82]. Thereby, Ad-VLPs are designed to carry compounds for anti-hepatocellular carcinoma such as the mRNA cap analog, DOX and Bleomycin (BLM) [41,42]. These VLP tropism strategies are vital for targeted delivery of drugs, and apply to other biomolecules as well.

## 3. The Strategies for Cargo Loading into VLPs

**Figure 2:**
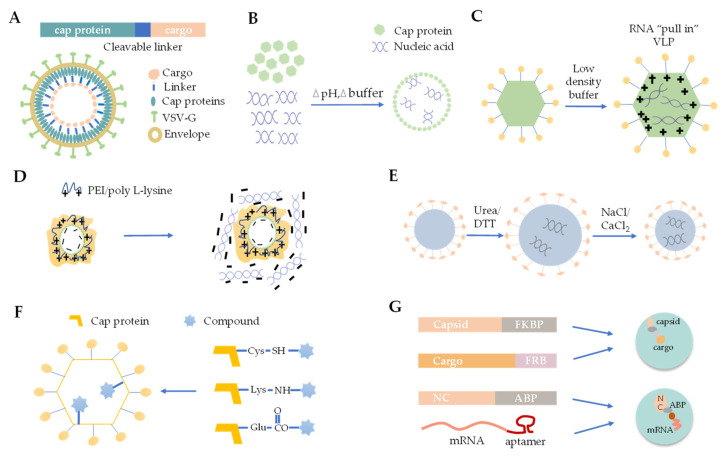
The strategies for cargos loading VLPs. (**A**) The strategy of foreign protein fusion. (**B**) De novo packaging of nucleic acids. (**C**) Osmotic shock. (**D**) Electrostatic adsorption. (**E**) Disassembly and reassembly. (**F**) Chemical linking. (**G**) Physical interaction between VLP and cargo.

Cargo loading is one of the most important steps for VLP-based delivery systems, and the approaches for loading cargo into VLPs include chemical, biological and physical strategies, which are summarized in Figure 2, and reviewed in detail.

### 3.1. Foreign Protein Fusion with VLPs 

The feature that many scaffold proteins for VLPs could maintain the capability of self-assembly when fused to foreign proteins or peptides enables direct fusion of protein cargo to the scaffold for cargo loading. Scaffold proteins of many viruses, such as MLV, HIV-1, and paramyxovirus have shown tolerance to cargo fusions. Protein cargo, depending on the conformation and structure of the scaffold-cargo fusion, could be caged inside of the particle or presented on the exterior surface of the particle, suitable for applications ranging from intracellular delivery of functional proteins or presentation of antigen proteins to immune cells. 

As each of the VLPs is assembled from multiple units of the scaffold protein, multiple number of cargo proteins could also be assembled into the same particle with the fusion strategy. Therefore, several types of proteins could be fused and delivered simultaneously, allowing for combinatorial delivery of several proteins and offering a powerful approach for combination and multivalent vaccine development. Chimeric VLPs which are generated by insertion of antigen peptides from other viruses or subtypes into VLPs have been widely used for new vaccine development [83,84,85]. In addition to antigen presentation, proteins with different features and functions, such as transcriptional factors, enzymes and antibodies, have been loaded and delivered by this strategy. Further, proteins with large size, like Cas9 nuclease or dCas9-fused base editors were successfully delivered into cells and showed their biological activities, demonstrating a general way for protein cargo loading [86,87,88,89]. At present, whether the scaffold protein interferes with the dynamics or functions of the cargo remains elusive. 

### 3.2. De Novo Packaging with Nucleic Acids

Nucleic acids are natural cargos of VLPs as infectious virions are formed by packaging of viral genomic DNA or RNA into the viral particles. Therefore, nucleic acids with certain sequences or structure properties could be recognized by the capsomere subunits and assembled into the VLP de novo. Mixing the subunit protein of some VLPs with certain nucleic acid molecules under certain conditions, the nucleic acid molecules could recruit capsomere proteins and facilitate VLP formation with encapsulated nucleic acids, shielding the desired nuclei acid molecules from the environment and allowing for intracellular delivery of the molecules [90].

### 3.3. Osmotic Shock 

Proteins, nucleic acids or drugs could also be loaded into the pre-formed VLPs, and one of the common ways is loading by osmotic shock. Pre-assembled VLPs are placed in buffers with low ionic strength, and the space distance between the surface subunits of VLPs would increase to accommodate the entry of cargo molecules such as nucleic acid molecules [91]. Physical interaction generated by the positively charged residues on the inner surface of VLPs can ‘pull’ nucleic acid molecules into VLPs [92]. 

### 3.4. Polymer Mediated Adsorption

Through a direct electrostatic interaction with the positively charged coatings or complexes such as PEI and poly L-lysine, the negatively charged nucleic acids can be loaded with VLPs [77]. Some viral capsid proteins contain negatively charged patches which could bind the polymers, cargo then could be loaded to the VLP-polymer complexes for delivery. Examples of HBc and AAV have been used to carry RNA or DNA for gene delivery systems, gene knockdown systems and vaccines [46,93,94,95]. 

### 3.5. Disassembly and Reassembly

Disassembly and re-assembly of the VLP in order to encapsulation different kinds of cargo can be achieved by chemical treatment with urea/NaCl or DTT/CaCl2 reagents [47]. The principle behind them is simple: urea and DTT are used as the denaturant and reductant to weaken the protein–protein interactions between capsomere subunits and loosen the VLP structure, allowing for cargo molecules (DNA or RNA) penetrate into the VLPs [96]. Subsequently, treatment with high concentration of NaCl or CaCl_2_ putatively enhances electrostatic repulsions between the protein monomers and facilitates reassembly of VLP containing nucleic acids [97,98]. 

### 3.6. Chemical Linking

Chemical linking is a classical approach for biomolecule coupling. On the one hand, cargo could be loaded by formation of covalent bonds between the VLP and cargo via chemical reactions. Multiple amino acid residues, such as lysine (amino group), cysteine (sulfhydryl group), aspartate and glutamate residues (carboxyl group) could be functionalized and form covalent bonds between the VLP and cargo via chemical reactions [77,99]. On the other hand, biochemical reactions could be used to couple protein cargo to the VLP, e.g., bacterial sortases have been used to ligate proteins with C-terminal LPXTGX to proteins with N-terminal oligoglycine/alanine [100]. With the quick development of click chemistry, which is simple and efficient, proteins and nucleic acids could be conjugated to azide or alkyne groups, then directly linked to the VLP surface using copper catalyzed or copper-free click chemistry [101,102,103,104].

### 3.7. Physical Interaction between VLP and Cargo

Several studies have found that the physical interaction force between capsids, protein and the others (CP−CP interaction) could drive the assembly of virions, and the packaging process of genome could also promote their assembly [105,106,107,108]. Accordingly, researchers inspired by these findings developed several strategies to incorporate cargo into the interior of VLPs [109,110]. One of such strategies is achieved by adding RNA packaging signal elements (like the psi packaging sequence) to cargos, the RNA packaging elements interact with capsid proteins and mediate the packaging of cargos. An example of this is that Segel et al. put the packaging signal sequences from PEG10 at both the 5’UTR and 3’UTR of mRNA cargos, successfully loading the mRNA of interest to VLPs [45]. Similarly, physical interaction between RNA aptamer and ABP could be also applied for RNA cargo loading. For instance, the NC protein of lentivirus-containing aptamers binds to cargo fusing with the ABP for Cas9 mRNA delivery [48]. Interaction between proteins could also be used for cargo loading, a good example is that the M protein of paramyxovirus can interact with cargo proteins which are attached to an appendage derived from the NP protein, and mediate packaging of the cargo protein [35].

## 4. The Manufacture and Application of VLPs 

### 4.1. Production, Stability and Immunogenicity of VLP for Cargo Delivery

Due to the diversity of the VLPs, multiple platforms based on bacterial-, yeast-, insect-, plant- and mammalian-cell expression systems have been developed for production of VLPs. Some VLPs with simple structures could assemble spontaneously when expressed in the prokaryotic or eukaryotic systems, or even formed in a cell-free system. Therefore, purification of these VLPs could be achieved by simple ultracentrifugation. But for more complicated VLPs, such as many enveloped VLPs, mammalian cell or baculovirus-insect expression systems are required to offer the lipid membrane for VLP formation. For some VLPs, such as VLPs obtained by non-secreted systems like bacteria, yeast and plant cells need to go through processes like cell lysis and purification, then assemble under suitable conditions to allow for formation of VLP structures. More specific details on production of VLPs have been summarized by recent review papers [76,111]. Accordingly, cargo could be loaded during the formation of VLP (such as cargos loaded by fusion strategy), or could also be loaded to purified VLP [33,112], depending on different loading strategies introduced in Section 3. The purified VLPs then could be analyzed by biophysical or biochemical approaches, such as EM or HPLC to characterize the homogeneity, cargo loading efficacy, stability etc., preparing for cargo delivery [113,114].

The stability of VLP is an important issue as the special nanostructure is the basis for successful loading and delivery of biomolecules. The stability of VLPs could be affected by temperature, pH, or ion concentration, for example [115], and it is also determined by the intrinsic property of the VLP. Several studies showed that VLPs generally have slightly lower thermostability than the intact virus [116,117], which might be because genomic nucleic acids are missing in VLPs therefore lacking the interaction between the genome and coating protein to stabilize the VLP. For VLPs loaded with cargo, the chemical and physical property of the cargo might also affect the stability or structure of the VLP. Therefore, due to their diversity, cargo might have distinct impacts on the stability of the VLPs. While cargo like nucleic acids which mimic the genome of viruses generally have no or even positive effects on the stability of VLPs, structure or stability of the VLP could also be impaired by the cargo. For example, TEM data showed that HBc VLP loaded with doxorubicin or geldanamycin did not present in a single morphology, some of the VLPs showed spherical or curved filamentous envelope structures rather than classical homogenous envelope structures, indicating the structure of VLP was affected by chemical cargo [118]. Efforts for VLP vaccine stabilizing such as adding a special peptide such as polyhistidine-peptide [119] to the VLP component might also work for delivery. As research on the structure and stability of cargo-loaded VLPs are quite limited currently, further studies on this basic property would help to extend the applicability of VLPs for delivery purposes. 

Notably, unlike the VLP for vaccine, which generally requires stable structure and good immunogenicity, ideal VLPs for other types of cargo delivery should have variable stability and low immunogenicity. On the one hand, the VLP should be stable enough to protect the cargo in vitro or in the body. On the other hand, once engulfed into the target cells, stability of the VLP could be a hurdle as the cargo needs to be released either naturally (such as endosome pH responsive) or in an artificially controlled way. Therefore, thermo-induced or near-infrared light responsive VLPs are designed, which could facilitate VLP disassembly and cargo release upon induction [120,121]. For in vitro application of VLPs, such as gene delivery into cultured cells, immunogenicity is not a problem, but for intracellular delivery of cargo in vivo, immunogenicity of the nanocarrier could be an important issue. Similar to other viral or chemical delivery vectors, the strategy for reducing immunogenicity of VLPs should be explored, such as modification of the VLP or substitution of the surface epitopes, utilizing VLPs with low antigenicity such as certain types of AAVs. In addition, VLPs assembled from endogenous viral proteins are also applied to avoid activation of the immune system [45]. 

### 4.2. VLP-Mediated Cargo Delivery in Biological and Biomedical Research

As mentioned above, the VLP has a wide range of applications such as vaccination, diagnostic imaging, and delivery of biomolecules and compounds. Plenty of VLP-based vaccines are in preclinical or clinical phases at present, which was summarized by a recent review from Mohsen and Bachmann [122], but VLPs serving as the vehicles for a cargo delivery system are mostly still in preclinical stages with a few exceptions. Here, we summarize the applications of diverse VLPs for intercellular delivery in biological and biomedical research, as shown in Table 2.

## 5. Conclusions and Future Perspectives

The first vaccine based on VLP was approved in 1986, and since then investigations of VLPs relative to vaccines have sprung up. With the advances of biological technologies in cutting-edge fields, VLPs have gradually become one of the most promising delivery vectors in biology and biomedicine areas, and multiple VLPs and various cargo loading methods have been developed for intracellular delivery of biomolecules and compounds.

Although with fascinating progress, many concerns and limitations need to be stressed. Importantly, further investigations on the tropism, target-cell specific VLPs and controlled release of the cargo are required for precise delivery of molecules into proper tissues and cells, which could significantly enhance the applicability of the method and still is one of the major weak points. 

Generalized cargo loading strategies and VLP platforms which could carry different types of cargo without rebuilding the system should be established. These efforts could lower the requirement for thoroughly optimizing cargo/VLP production conditions, resulting in a more practicable technology. For example, a generalized mRNA delivery platform for antigen presenting would greatly help to develop new RNA vaccines in case of a new virus emerging. For RNA delivery and RNA vaccine development, VLPs stabilizing the cargo RNAs would be of special interest as the current lipid-based RNA vaccine requires strict conditions during transportation and distribution, which increases the cost and makes it impracticable for remote areas. 

As virus generated delivery tools, the immunogenicity of VLPs could not be neglected, undesired immune response or preexisting neutralizing antibodies could lead to failure of molecular delivery or gene therapy. Therefore, how to escape from the surveillance of the immune system and reduce immunogenicity to avoid adverse cellular responses would be one vital topic for future research. Endogenous virus-derived VLPs [45] or artificially designed VLPs could be solutions for reducing potential immunogenicity of the VLPs, but extensive investigations are required to overcome the limitation. With those efforts, delivery with VLPs would be more practicable in both basic research and also for clinical applications.

## Figures and Tables

**Table 1:** VLPs for intracellular delivery of biomolecules and compounds.

VLP Origin	Components of the VLP	Cargo Packaging Strategies	Specialties of the Delivery System	VLP Production Systems	Refs.
Murine leukemia virus (MMLV or FMLV)	Gag, Gag-Pol, VSV-G	Foreign protein fusion to VLP	Enveloped virus; protein delivery; allow for loading large molecular proteins such as Cas9	HEK-293T producer cells Gesicle 293T producer cells	[29,31,32,33,40]
Avian sarcoma leukosis virus (ASLVs)	Gag, VSV-G	Foreign protein fusion to VLP	Enveloped virus; protein delivery	Mammalian cell expression system	[30]
Paramyxovirus (PIV5, Nipah)	NP, M, glycoprotein	Physical interaction between cargo and scaffold proteins	Adaption to suspension cell cultures for large-scale production; enveloped virus; few limitations in the size of cargos	Mammalian cell expression system	[35]
Human Immunodeficiency Virus-1 (HIV-1)	Capsid p24 protein, Nef7, VSV-G	Foreign protein fusion to VLP	Enveloped virus; more safety compared with traditional treatments of cancer	293T producer cells	[36]
Bluetongue virus	VP3, VP7, VP5 and VP2	Foreign protein fusion to VLP	Non-enveloped virus; efficiently kill tumor cells	Plant expression system	[34]
Adenovirus	Penton-dodecahedron (Pt-Dd)	Conjugation reaction	Non-enveloped virus; chemical linking does not affect the VLP capability to enter cells, even easier internalization; low immune response	Baculovirus-insect cell expression system	[41,42]
Artificial proteins	C-S_10_-K_12_ protein	Electrostatic adsorption	Non-enveloped artificial VLP; pronounced physical stability; scarcely existing cytotoxicity and hemolysis for target cells	Pichia pastoris expression system	[43,44]
Endogenous retrovirus (PEG10)	VSV-G, PEG10	Physical interaction between mRNA and VLP	VLPs derived from a full human system for mRNA delivery	HEK 293T cells expression system	[45]
AAV2	PEI, Cap (Vp1, Vp2, Vp3)	Electrostatic adsorption	Non-enveloped virus; no pronounced cytotoxicity; engineered for targeting	Baculovirus-insect cell expression system	[46]
HBV	Hepatitis B virus core protein (HBc)	Disassembly and reassembly; osmotic shock	Enveloped virus; compatible for siRNA delivery; good biocompatibility; diminish a strong immune response; good stability in serum	*E. coli* expression system	[47]
Lentivirus	1. Gag (NC, MA, CA) 2. Gag-pol, Gag, VSV-G	Physical interaction between VLP capsid proteins and cargos	Enveloped virus; more highly efficient in genome editing	HEK 293T producer cells	[48,49]
Foot-and-mouth disease virus (FMDV)	VP0, VP1 and VP3	Covalent connection	Non-enveloped virus; targeted delivery to tumor cells avoids side effects in normal tissues	*E. coli* expression system	[50]
Rotavirus	VP6	Covalent connection	Non-enveloped virus; DOX releases at low pH preventing leak in the bloodstream	*E. coli* expression system	[51]
Rous sarcoma virus (RSV)	Gag	Physical method (electroporation)	Enveloped virus; same amount of DOX loading into VLP is more efficient for killing cells	Silkworm larvae expression system	[52]
Porcine parvovirus	VP2	Covalent connection	Non-enveloped virus; TK peptide is a dual-functional ligand	Baculovirus-Sf9 insect cell expression system	[53]
Bacteriophage (MS2, Qβv)	SP94, H5WYG, PEG, Coat protein dimers	Disassembly and reassembly; physical interaction between VLP and cargo	Keep good stability in different conditions; various cargos can be packaged into VLP (RNA, DNA, proteins, compounds)	*E. coli* expression system	[54]
Bacteriophage (PP7, MS2)	Coat protein dimers, TAT peptide	Physical interaction between bacteriophage-like particles and miRNA linked to stem-loop	Heat-resistant at high temperature (≤ 60℃)	*E. coli* expression system	[55,56]
Bacteriophage P22	Scaffold proteins, Capsid proteins	Foreign protein fusion to VLP	Keep good stability and protect cargo from degradation	*E. coli* expression system	[57]
Bacteriophage Qβ	Capsid proteins	Covalent connection	Macrophage can be activated by polyvalently displaying macrolides to the surface of Qβ VLPs	*E. coli* expression system	[58]

VSV-G: vesicular stomatitis virus glycoprotein; NC: nucleocapsid; MA: matrix protein; CA: capsid; DOX: doxorubicin; C-S_10_-K_12_: hydrophilic random coil polypeptide(C), silk protein-like midblock S10 which is (GAGAGAGQ)10, oligolysine (K12); PEI: polyethylenimine; VP: viral protein; SP94: SFSIIHTPILPL peptides targeting hepatocellular carcinoma; H5WYG: fusogenic peptides that promote the VLP to escape from endosomal pathway; PEG: reduce nonspecific interactions and immunogenicity of VLP; TAT peptide: transactivated transcription peptide with cell-penetration ability.

**Table 2:** Applications of VLP-mediated cargo delivery.

VLP Origin	Cargo	Applications	Testing	Targeting Strategies	Refs.
Murine leukemia virus (MLV)	1. Flp recombinase 2. GFP 3. Nuclear transcription factors 4. Bacterial toxin/anti-toxin system	Gene recombination, cell differentiation, cell death	Murine iPSCs; mouse embryonic fibroblast cell line (SNL cells); HeLa MCAT cell line	Pseudotyped VSV-G envelope; EA6 envelope	[29,31]
Avian sarcoma leukosis virus (ASLVs)	1. Cre recombinase 2. Human caspase-8 3. Active pro-drug enzymes (Fcy and Fur)	Gene recombination, cancer treatment	PC3 cells	VSV-G envelope; ligand/receptor mediated delivery (NA-IFN-γ ligand or HA-TNF ligand)	[30]
Friend murine leukemia virus (FMLV)	1. Cas9-sgRNA ribonucleoproteins 2. Cas9 fusion complexes	Gene editing, gene knock-in, transcriptional activation, transgenic animals	Primary cells (hiPSCs, HSCs, mouse bone marrow); mouse embryos; liver of injected mice	VSV-G envelope; BaEV pseudotyped envelope	[32]
Friend murine leukemia virus (FMLV), Moloney murine leukemia virus (MMLV)	ABE8e (base editor)	Gene(base)-editing, genetic disorders treatment	HEK293T cells, primary human and mouse cells (primary human T cells, primary human/mouse fibroblasts), different organs (liver, brain, eye of mouse) in mouse	VSV-G envelope	[33]
Paramyxovirus (PIV5, Nipah)	1. Rluc 2. GFP 3. Superoxide dismutase 4. Cre recombinase	Restore oxidative stress	A549 cells Reporter cells	Tropism of natural virus (such as target sialic acid surface receptors, ephrin-B receptors)	[35]
Human Immunodeficiency Virus-1 (HIV-1)	1. GFP 2. HSV-1 thymidine kinase	Cell suicide therapies	CEM-ss cells and human primary macrophages	VSV-G envelope	[36]
Bluetongue virus	HSV-1 thymidine kinase	Anti-tumor treatment	Human glioblastoma derived cells	Natural tropism	[34]
Adenovirus	DOX, Bleomycin (BLM)	Anti-hepatocellular carcinoma	Neoplastic cells	Targeting peptides	[41,42]
Artificial proteins	mRNA	A therapeutic agent	HeLa and HEK293 cells	Not mentioned	[43]
Endogenous retrovirus (PEG10)	Cre mRNA and SpCas9 mRNA/sgRNA	Gene therapy	Reporter N2a cells HEK293FT cells	VSV-G envelope; endogenous MmSYNA envelope	[45]
AAV2	siRNA	Breast cancer treatment	MCF-7 breast cancer cell	Not mentioned	[46]
HBc	siPLK1	Cancer treatment	Cancer cells Mouse tumor model	Ligand/receptor mediated delivery (HER2)	[47]
Lentivirus	1. Cas13 RNP 2. SpCas9 mRNA/sgRNA	Anti-virus infection Gene Knockout	Primary human cells	VSV-G envelope	[48,49,70]
Bacteriophage (MS2, Qβ)	siRNA, chemotherapy drugs (DOX, 5-FU, cisplatin), ricin toxin A-chain	Cell apoptosis; cancer treatment	Human hepatocellular carcinoma cell line (HCC)	SP94	[54]
Bacteriophage (PP7, MS2)	MicroRNA (pre-miR-23b, miR-122)	Hepatoma treatment	hepatoma SK-HEP-1 cells, hepatocarcinoma cell lines	Cell-penetrating peptide (TAT peptide)	[55,56]
Bacteriophage P22	Cas9/sgRNA	Gene therapy	dsDNA cleavage assay	Not mentioned	[57]
Bacteriophage Qβ	Macrolide antibiotics (azithromycin and clarithromycin)	Antimicrobial infection	RAW 264.7 macrophage cells, lungs tissue in mice	Azithromycin directs the VLPs to the lungs	[58]
Foot-and-mouth disease virus (FMDV)	DOX	Tumor treatment	HeLa cells	RGD motif	[50]
Rotavirus	DOX	Hepatoma treatment	HepG2 cell	Lactobionic acid	[79]
Rous sarcoma virus (RSV)	DOX	Colon carcinoma treatment	LS174T cell	hCC49 antibody scFv	[52]
Porcine parvovirus	DOX	Colorectal cancer treatment	Caco-2 cell and HUVEC cell	TK peptide	[53]

EA6-3X: a modified MLV EA6 strain env; BaEV: baboon endogenous virus; NA: influenza neuraminidase; HA: hemagglutinin; siPLK1: siRNA of polo-like kinase 1 gene; HER2: human epidermal growth receptor 2; SpCas9: Streptococcus pyogenes Cas9; ABE8e: adenine base editor; DOX: doxorubicin; BLM: Bleomycin; TK peptide: TWYKIAFQRNRK peptide, a bi-functional targeting ligand; hCC49 antibody: humanized CC49 antibody; scFv: single chain fragment variable; 5-FU: 5-fluorouracil, anti-tumor drugs; SP94: peptide with the sequence SFSIIHTPILPL which targets hepatocellular carcinoma (HCC).
